# Cell Fusion along the Anterior-Posterior Neuroaxis in Mice with Experimental Autoimmune Encephalomyelitis

**DOI:** 10.1371/journal.pone.0133903

**Published:** 2015-07-24

**Authors:** Sreenivasa R. Sankavaram, Mikael A. Svensson, Tomas Olsson, Lou Brundin, Clas B. Johansson

**Affiliations:** 1 Center for Molecular Medicine, Karolinska University Hospital, Stockholm, Sweden; 2 Department of Clinical Neuroscience, Karolinska Institutet, Stockholm, Sweden; 3 Public Dental Service at Gällö, Jämtland Härjedalen County Council, Gällö, Sweden; Witten/ Herdecke University, GERMANY

## Abstract

**Background:**

It is well documented that bone marrow-derived cells can fuse with a diverse range of cells, including brain cells, under normal or pathological conditions. Inflammation leads to robust fusion of bone marrow-derived cells with Purkinje cells and the formation of binucleate heterokaryons in the cerebellum. Heterokaryons form through the fusion of two developmentally differential cells and as a result contain two distinct nuclei without subsequent nuclear or chromosome loss.

**Aim:**

In the brain, fusion of bone marrow-derived cells appears to be restricted to the complex and large Purkinje cells, raising the question whether the size of the recipient cell is important for cell fusion in the central nervous system. Purkinje cells are among the largest neurons in the central nervous system and accordingly can harbor two nuclei.

**Results:**

Using a well-characterized model for heterokaryon formation in the cerebellum (experimental autoimmune encephalomyelitis - a mouse model of multiple sclerosis), we report for the first time that green fluorescent protein-labeled bone marrow-derived cells can fuse and form heterokaryons with spinal cord motor neurons. These spinal cord heterokaryons are predominantly located in or adjacent to an active or previously active inflammation site, demonstrating that inflammation and infiltration of immune cells are key for cell fusion in the central nervous system. While some motor neurons were found to contain two nuclei, co-expressing green fluorescent protein and the neuronal marker, neuron-specific nuclear protein, a number of small interneurons also co-expressed green fluorescent protein and the neuronal marker, neuron-specific nuclear protein. These small heterokaryons were scattered in the gray matter of the spinal cord.

**Conclusion:**

This novel finding expands the repertoire of neurons that can form heterokaryons with bone marrow-derived cells in the central nervous system, albeit in low numbers, possibly leading to a novel therapy for spinal cord motor neurons or other neurons that are compromised in the central nervous system.

## Introduction

Cell fusion between cells of the same developmental origin is a natural, biological process that is essential during e.g. fertilization, myotube formation, inflammation, and homeostasis maintenance of the skeleton [1–4]. Further, several groups have recently demonstrated that cells from different developmental origins can yield lineages that are capable of gaining access to diverse tissues including skeletal muscle, liver, heart, intestine and cerebellum and contribute to the function of these tissues by cell fusion [5–14].

We have previously demonstrated that cells from different developmental origins, e.g. bone marrow-derived cells (BMDCs) and Purkinje cells, fuse and form stable, functional binucleate heterokaryons in the cerebellum; this natural phenomenon is greatly enhanced by inflammation [15–18]. In the rodent model for multiple sclerosis (MS), experimental autoimmune encephalomyelitis (EAE), massively increased fusion rates occur in the cerebellum as compared to non-inflamed animals. This finding has recently been extended to humans, with the demonstration of heterokaryon formation in the cerebellum of MS patients [19].

Given that BMDCs fuse with cells of a different origin after transplantation to foreign tissue, it has been speculated that cell fusion may be an attractive approach to restore function to diseased tissue [20,21]. Gibson et al. [22] pioneered the regenerative aspects of cell fusion using muscular dystrophic mutant mice (a mouse model of Duchenne’s muscular dystrophy). After dermal fibroblasts were transplanted to mutant mice, fibroblasts fused with myotubes and partially protected the mice against muscle dysgenesis [22]. A further example where cell fusion can save cells relates to tyrosinemia, a metabolic and lethal recessive liver disease. In mice with tyrosinemia, transplantation of wild-type BMDCs restored liver function by cell fusion and prevented death, indicating that cell fusion can have beneficial effects [11,23]. Cell fusion as a therapeutic approach in regenerative medicine may be particularly attractive in the CNS, where the cellular architecture cannot only be complex, but crucial to functionality. A cerebellar Purkinje cell, for example, has a massive dendritic network of up to 100,000 synaptic connections to regulate intricate motoric functions, and spinal cord motor neurons must extend and correctly wire meter-long axons. Replacing such intricate cells by transplantation of, for instance, stem cells would require *de novo* integration in a complex circuitry to mimic functionality, however, the feasibility of such an approach remains to be shown. By inducing the fusion of donor BMDCs to damaged recipient cells that are already located and integrated in the tissue, such problems may be avoided [20]. In the CNS, transplantation of mesenchymal stem cells (MSCs) into the cerebellum of a mouse model of a metabolic disease, Niemann-Pick disease type C (NPC1), led to fusion of MSCs with compromised Purkinje cells *in vivo*, partially correcting the impaired sphingolipid metabolism associated with NPC1 transgenic mice, resulting in a mild motoric improvement [24,25]. Cell fusion of BMDCs to cells of the CNS is not limited to rodents, but has also been observed in humans [13,19]. These findings may open up new possibilities in regenerative medicine using a yet unknown, natural cell fusion mechanism to introduce not only healthy genomic DNA (gDNA) but also organelles, such as mitochondria, into compromised cells and consequently restore these cells.

Our previous studies have demonstrated that inflammation is a key player in cell fusion processes and greatly enhances the fusion rates in the cerebellum [15]. In the absence of inflammation very few heterokaryons are detected. Moreover, in the brain, fusion of BMDCs appears to be restricted to Purkinje cells of the cerebellum [5,13–18]. This may be due to (i) their large size, enabling them to more easily harbor a second nucleus, and (ii) their complexity, as neural stem cells (NSCs), where they have been described in the subventricular zone and hippocampus, which means they may not be appropriate in replacing degenerating Purkinje cells. Here, we hypothesize that under the appropriate (inflammatory) circumstances, BMDCs may also be able to fuse with other neurons, in particular large and complex ones. Therefore, we examined the formation of heterokaryons in the entire anterior-posterior neuroaxis: retina, olfactory bulb, cortex, hippocampus, cerebellum, and the spinal cord. We demonstrate for the first time that cell fusion is not a feature unique to the interaction between BMDCs and Purkinje cells of the cerebellum, but that BMDCs can fuse, albeit in low numbers, with motor neurons in the adult spinal cord in the EAE mouse model of multiple sclerosis. Cell fusion predominately occurred in areas of major inflammation and infiltration of immune cells in the cerebellum and the spinal cord. We hypothesize that cell fusion and the formation of stable binucleate heterokaryons can also restore damaged neurons outside the cerebellum, and may eventually lead to state-of-the-art therapies for metabolic and neurodegenerative diseases.

## Material and Methods

### Animals

GFP^+^CD45.1 C57BL/6 and C57BL/6 mice were either bred in-house or obtained from Jackson Laboratory. Mice were maintained in a pathogen-free environment with free access to water and food in a 12 h light/dark environment.

### Ethics statement

All animal experiments were approved by the Administrative Panel on Laboratory Animal Care, APLAC protocol ID number 13226 at the Stanford University School of Medicine and the IACUC approval number is A3213-01.

### Bone marrow transplantation

All mice received bone marrow transplants at 8 weeks of age, as described previously [10,15].

### Experimental autoimmune encephalomyelitis induction (EAE)

EAE was induced in 14-week-old female C57BL/6 mice previously transplanted at 8 weeks of age with bone marrow from 12-week-old GFP^+^CD45.1 C57Bl/6 mice as described previously [15]. Briefly, mice were immunized with 100 μg of myelin oligodendrocyte glycoprotein (MOG) p35–55 in the flanks. MOG was dissolved in phosphate-buffered saline (PBS) and mixed with complete Freund's adjuvant containing 2 mg/ml of heat-killed Mycobacterium tuberculosis H37Ra (Difco Laboratories). On the day of immunization and 48 h later, mice were injected intravenously with Bordetella pertussis toxin (75 ng in PBS). Mice were examined daily for clinical signs of EAE and scored on a scale of 0–5, as described previously [15]. The mice were perfused with PBS followed by ice-cold paraformaldehyde (4%) in phosphate buffer, as described previously [15] 50 days after the EAE induction.

### Perfusion of mice and collection of tissue

Mice were killed and perfused at various times after bone marrow transplantation, as described previously [26]. Briefly, mice were anaesthetized (ketamine, 120 mg/kg + xylazine, 10 mg/kg, intraperitoneally) and immediately perfused with PBS followed by ice-cold 4% paraformaldehyde in phosphate buffer, and cryoprotected in sucrose/phosphate buffer solution overnight. Cerebellum and spinal cord were sectioned at 60 μm for antibody staining using a sliding microtome (SM2000R; Leica). Eyes, olfactory bulb and cerebrum were sectioned at 30 μm using a cryostat microtome (Leica CM 3000). The sciatic nerve was sectioned at 16 μm using a cryostat.

### Immunofluorescence and imaging

Free-floating cerebellar and spinal cord sections were incubated overnight at 4°C with rabbit anti-green fluorescent protein (GFP) (1:2000; Molecular Probes) and mouse anti-Calbindin D-28K (1:1000; Sigma) or either mouse anti-neuron-specific nuclear protein (NeuN) (1:500; Millipore), or goat anti-choline acetyltransferase (ChAT) (1:100; Millipore) respectively. Histology slides of the eye, olfactory bulb and cerebrum were stained with rabbit anti-GFP (1:2000; Molecular Probes), mouse anti-NeuN (1:500; Millipore) and mouse anti-beta-III-tubulin (1:1000; Millipore) or goat anti-GFP (1:1000; Rockland) and rabbit anti-microtubule-associated protein 2 (Map2) (1:500; Millipore). Primary staining was followed by washing and incubation with appropriate secondary antibodies (goat anti-mouse Alexa 546; 1:1000 and goat anti-rabbit Alexa 488; 1:1000, or donkey anti-goat Alexa 488; 1:1000 and donkey anti-rabbit Alexa 546; 1:1000, or donkey anti-rabbit Alexa 488; 1:1000 and donkey anti-goat Alexa 546; 1:1000, all Molecular Probes) overnight at 4°C. Nuclei were stained with To-Pro-3 or Hoechst 33342 (all 1:3000; Molecular Probes) and sections were mounted on Superfrost Plus slides (Thermo Scientific) with ProLong Gold anti-fading mounting medium (Molecular Probes) [15]. Images were captured using either a Zeiss LSM 510 or Leica SP2/SP5 confocal microscope. We used pseudocoloring of nucleic staining in red or blue to enhance visibility in some images. Schematic illustrations of the pictures were drawn using Adobe Photoshop tools, in enhanced brightness and contrast.

### Quantification of heterokaryons along the neuroaxis

The entire retina, olfactory bulb, 3 mm of the cerebrum (spanning the motor cortex, lateral ventricles and the hippocampus), the entire cerebellum and part of the spinal cord at level thoracic level 1–2 and lumbar level 3–4 were examined and scored for heterokaryons. Heterokaryons were identified on the basis of morphology (Purkinje cells and motor neurons) and co-expression of GFP and Calbindin in the cerebellum (Purkinje cells), or GFP and Map2, beta-III-tubulin, NeuN and ChAT, in the olfactory bulb, cerebrum and the spinal cord, respectively. In the spinal cord, we quantified the presence of heterokaryons in areas of strong infiltration of immune cells (spinal cord at thoracic level 1–2 and lumbar level 3–4), at a rate of 20 sections per animal, in five EAE immunized mice and in corresponding sections of three healthy controls. We quantified the presence of heterokaryons across the entire cerebellum, comparing the vermis (±1.5 mm lateral of the midline) and the lateral hemispheres in seven EAE immunized mice and in three healthy controls.

## Results and Discussion

### Absence of cell fusion in non-inflamed areas of the central nervous system

It has been well demonstrated that BMDCs can fuse with Purkinje cells in the cerebellum under certain conditions [5,15,16,18]. One such condition is inflammation that enhances the cell fusion frequency 100-fold in the cerebellum. To identify whether BMDCs can fuse with neurons in other parts of the CNS, we used EAE as a well-established model of neuroinflammation, which induces clinical symptoms of multiple sclerosis. Mice previously transplanted with bone marrow from GFP-expressing donors, and immunized with myelin oligodendrocyte glycoprotein (MOG) to induce EAE were scored, as described previously [27] on a daily basis following the same standard protocol as in Johansson et. al. [15], starting at day 10 post-immunization. Mice were sacrificed 50 days post-immunization. All analyzed experimental mice (n = 7) alternated between score 2–3 for 30–40 days; healthy controls (n = 3) showed no impairment and thus scored 0.

Sections from different parts of the CNS, including the retina, olfactory bulb, motor cortex, hippocampus and the lateral ventricles from EAE affected mice were analyzed. No infiltration of GFP-labeled BMDCs could be seen in any retinal sections. Further, in none of the seven immunized and analyzed mice could we see a major infiltration of leukocytes in the cerebrum that was comparable to EAE lesions located in the cerebellum. In the motor cortex, along the lateral ventricles and in the hippocampus, the majority of GFP-labeled cells were restricted to blood vessels (data not shown).

Low numbers of GFP-labeled infiltrating BMDCs were detected in the olfactory bulb (S1A Fig). The majority of these GFP-labeled cells were infiltrating leukocytes, morphologically resembling macrophages (S1B Fig). The detected GFP-labeled peripheral blood-derived monocytes in the CNS are likely a consequence of the irradiation and bone marrow transplantation itself, since controls (non-immunized but irradiated and transplanted) showed similar numbers of infiltrating BMDC (data not shown). Only few GFP-labeled cells in the olfactory bulb were co-labeled with neuronal marker Map2 (S1C Fig). We could not reveal that these double-labeled cells were binucleated.

### Heterokaryons are detected in areas of immense infiltration of immune cells

**Fig 1:**
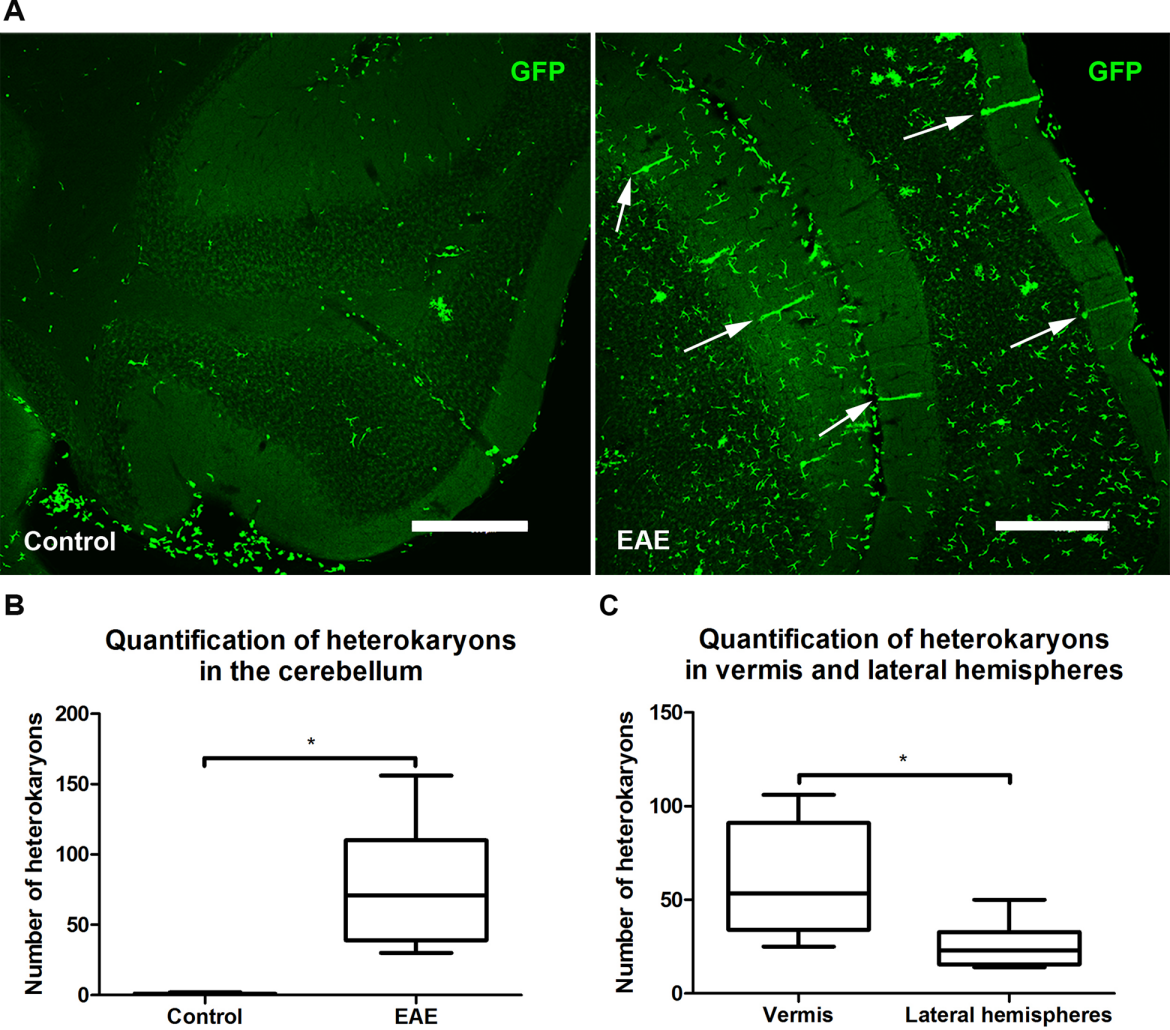
Immune cell infiltration and heterokaryon formation in EAE mouse cerebella. (A) Comparison between coronal sections of cerebella from Control (left) and EAE immunized mice (right). There is a higher number of GFP-labeled infiltrating bone marrow-derived cells and Purkinje heterokaryons (arrows) in EAE affected mice as compared to control animals. Scale bar 300 μm. (B) Quantification of Purkinje heterokaryons in Control (n = 3; 1.0 ± 0.6) and EAE (n = 7; 77.7 ± 16.7) shows a significant difference (p = 0.0167, Mann-Whitney-Wilcoxon test). (C) In EAE, more heterokaryons were located in the vermis (54.4 ± 12.0) than in the lateral hemispheres (n = 7; 23.3 ± 5.0) (n = 7, p = 0.0313).

The absence of massive immune cell infiltration to the cerebrum could explain the lack of heterokaryons. Hence, to confirm that BMDCs can form heterokaryons in our EAE model, we analyzed the cerebellum, typically affected by an ongoing EAE inflammation. In EAE affected mice, we detected varying degrees of infiltration of peripheral blood leukocytes and a significantly higher number of cerebellar heterokaryons (n = 7; 77.7 ± 16.7), than in non-immunized controls (n = 3; 1.0 ± 0.6; p = 0.0167, Mann-Whitney-Wilcoxon test, Fig 1A and 1B). Heterokaryons were exclusively detected in areas of massive immune cell infiltration as previously tissue demonstrated [15].

Anatomically, the cerebellum is divided into the two lateral hemispheres, and the vermis in the middle. It is known that in older individuals, Purkinje cells are lost in the anterior part of the cerebellum, while Purkinje cells in the hemispheres are less affected by age [28]. In our EAE animals, the lesion had a tendency to be localized to the vermis, and also significantly more heterokaryons were localized in the vermis (54.4 ± 12.0) as compared to lateral hemispheres (23.3 ± 5.0) (n = 7, p = 0.0313, Wilcoxon signed rank test), Fig 1C.

### BMDCs fuse and form heterokaryons with spinal cord motor neurons

**Fig 2:**
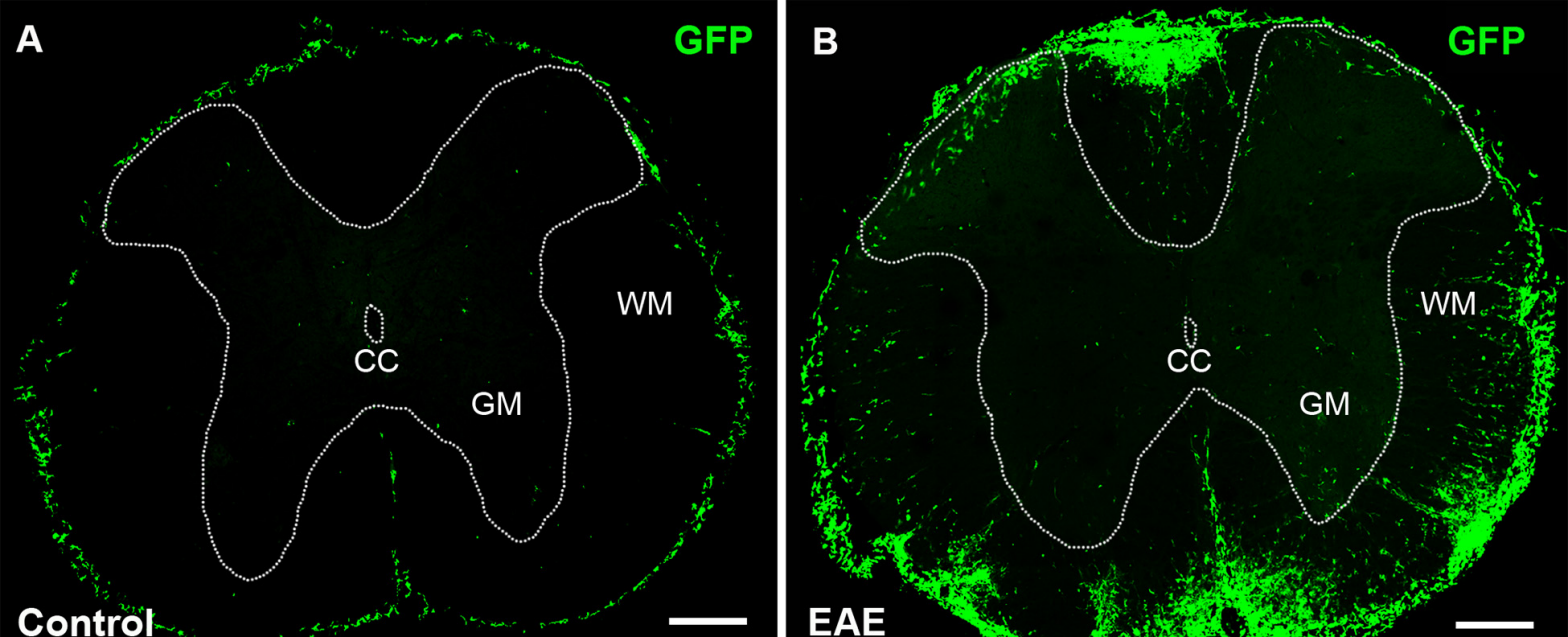
Immune cell infiltration in the spinal cord of Control and EAE affected mice. Coronal spinal cord sections showed little infiltration of GFP-labeled bone marrow-derived cells (green) in control (left) animals, while infiltration in EAE immunized animals (right) was prominent. Scale bar 300 μm.

**Fig 3:**
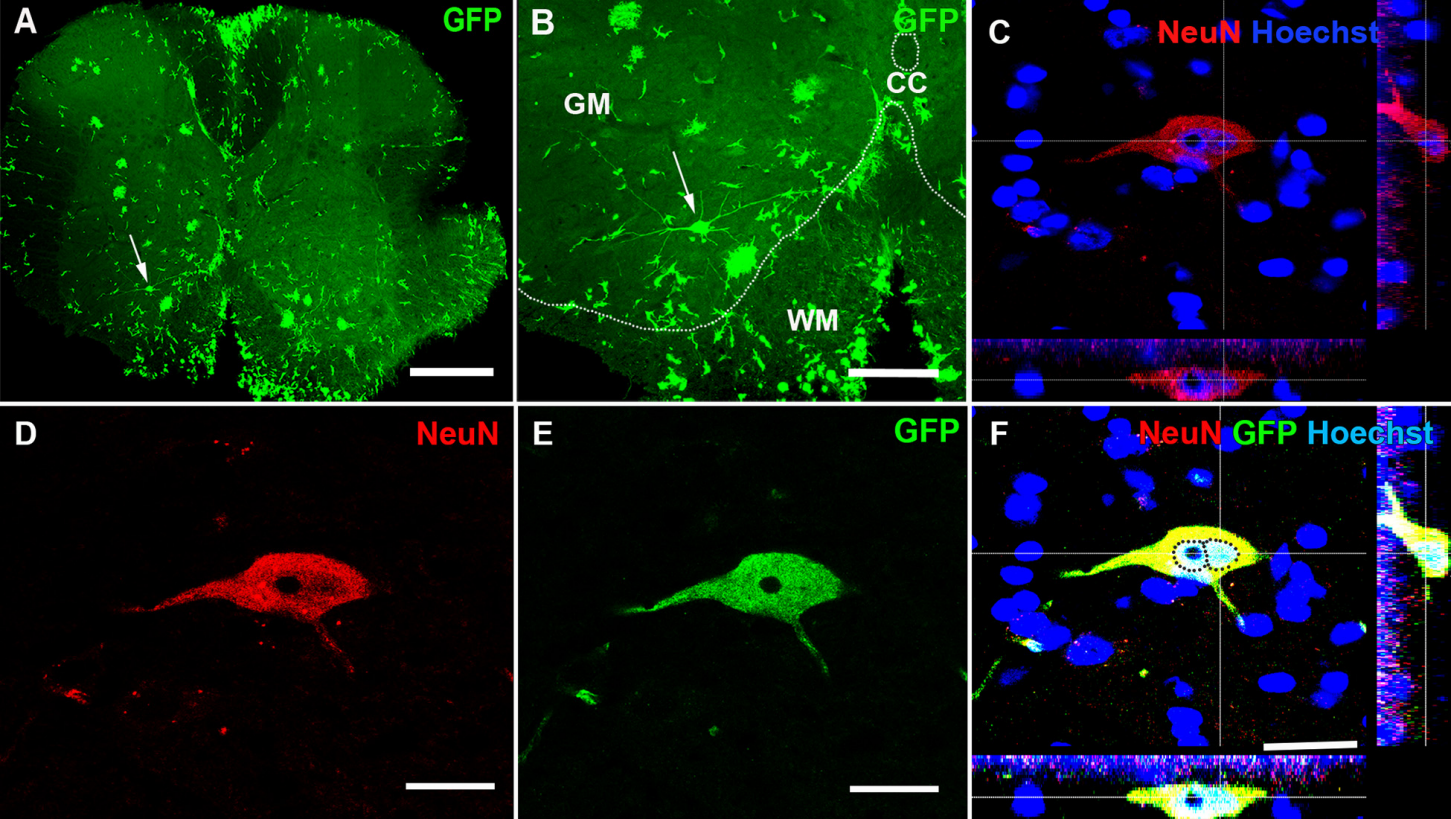
Formation of a heterokaryon in the spinal cord. (A) A motor neuron that is present in the ventral horn of the spinal cord expresses GFP (arrow) as a result of fusion between a GFP expressing bone marrow-derived cell and a motor neuron. The arrow shows a single GFP-labeled motor neuron in the ventral horn of the spinal cord. Scale bar 300 μm. (B) Higher magnification of a spinal cord GFP-labeled motor neuron shown in (A). Scale bar 150 μm. (C-E) Z-stack images of the GFP-labeled motor neuron shown in (A) and (B). (D-E) Immunohistochemistry demonstrating that the GFP-labeled motor neuron co-expresses GFP and NeuN. (F) Triple staining of the same motor neuron, NeuN (red), GFP (green) and Hoechst (blue). Two nuclei (Hoechst, blue) are present in the same cell, marked with dotted circles thus it is a heterokaryon. Scale bar 25 μm.

**Fig 4:**
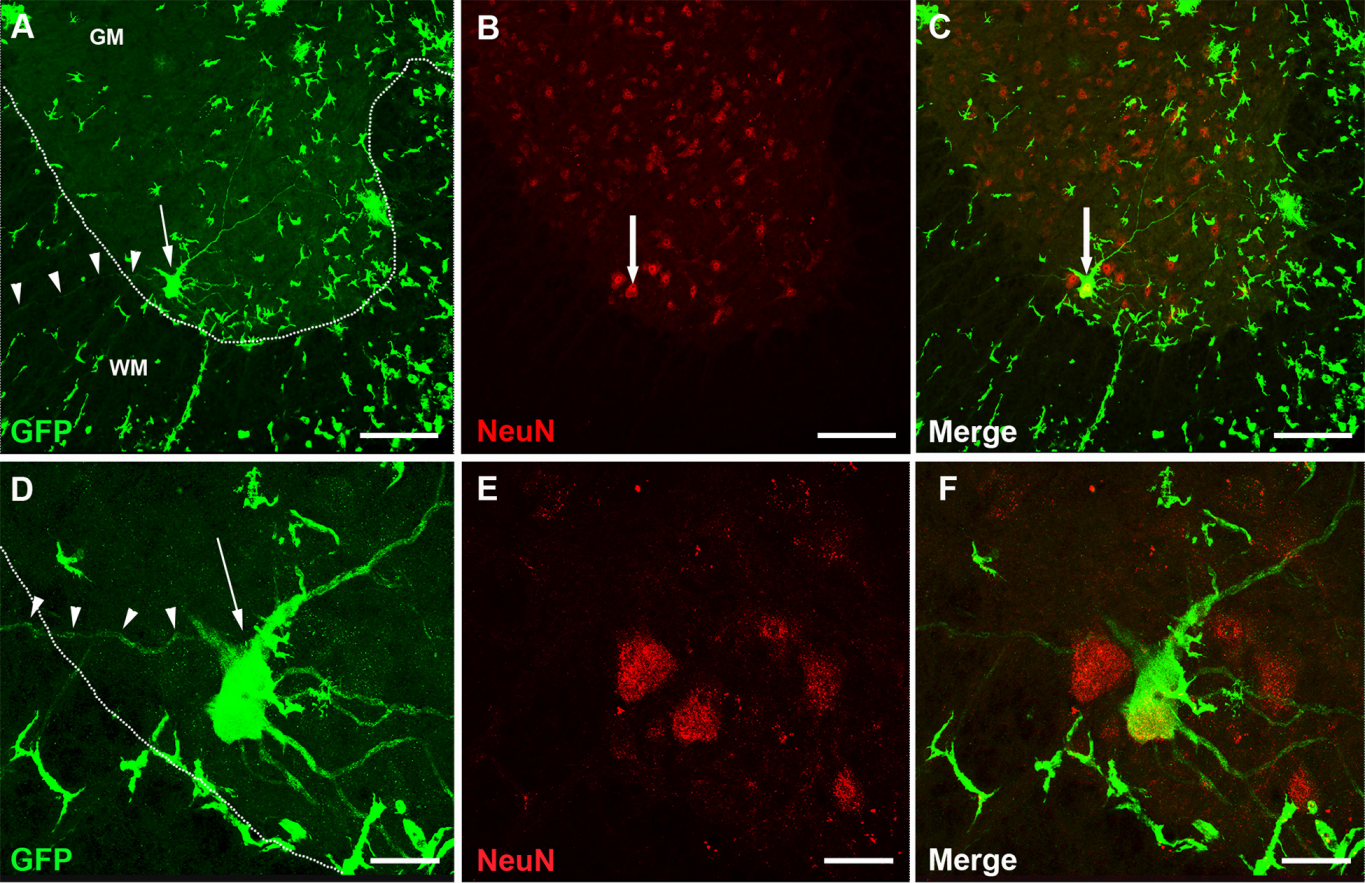
GFP-labeled spinal cord motor neuron co-expressing NeuN. (A) GFP-labeled (green) ventral horn motor neuron (arrow) extending a single axon from the grey matter (GM) to the white matter (WM) (see arrowheads). (B-C) This GFP-labeled motor neuron co-expresses NeuN. (D-F) Higher magnification of the motor neuron in B-C. Scale bar (A-C) 150 μm, (D-F) 25 μm.

Purkinje cells are among the largest cells in the CNS and we hypothesize that it is only large cells that can harbor two nuclei and retain a stable functional identity post fusion. Other large neurons in the CNS are pyramidal cells in the cerebral cortex and motor neurons in the spinal cord. Therefore, we speculate that BMDCs might be able to fuse with motor neurons, forming stable heterokaryons. We analyzed different parts of the spinal cord at thoracic level 1–2 and lumbar level 3–4, particularly areas with prominent infiltration of immune cells (Fig 2A and 2B). Excitingly, we found several GFP-labeled spinal cord motor neurons, indicating that fusion between GFP-labeled BMDCs and motor neurons had taken place (Fig 3A–3F and S2–S4 Figs). These GFP-labeled motor neurons were primarily located in the ventral horns as well as the lateral column of the spinal cord; almost exclusively in or adjacent to major immune cell infiltration (S3 Fig). Some GFP-labeled motor neurons were binucleated (Fig 3A–3F and S4A–S4H Fig) and some were positive for the neuronal marker NeuN (Fig 4A–4F, S5A–S5H and S6A–S6D Figs). Sciatic nerve analysis of EAE affected mice also supported the fusion hypothesis between motor neurons and GFP-labeled BMDCs. Several GFP-labeled fibers were detected along the sciatic nerve (S7 Fig).

**Fig 5:**
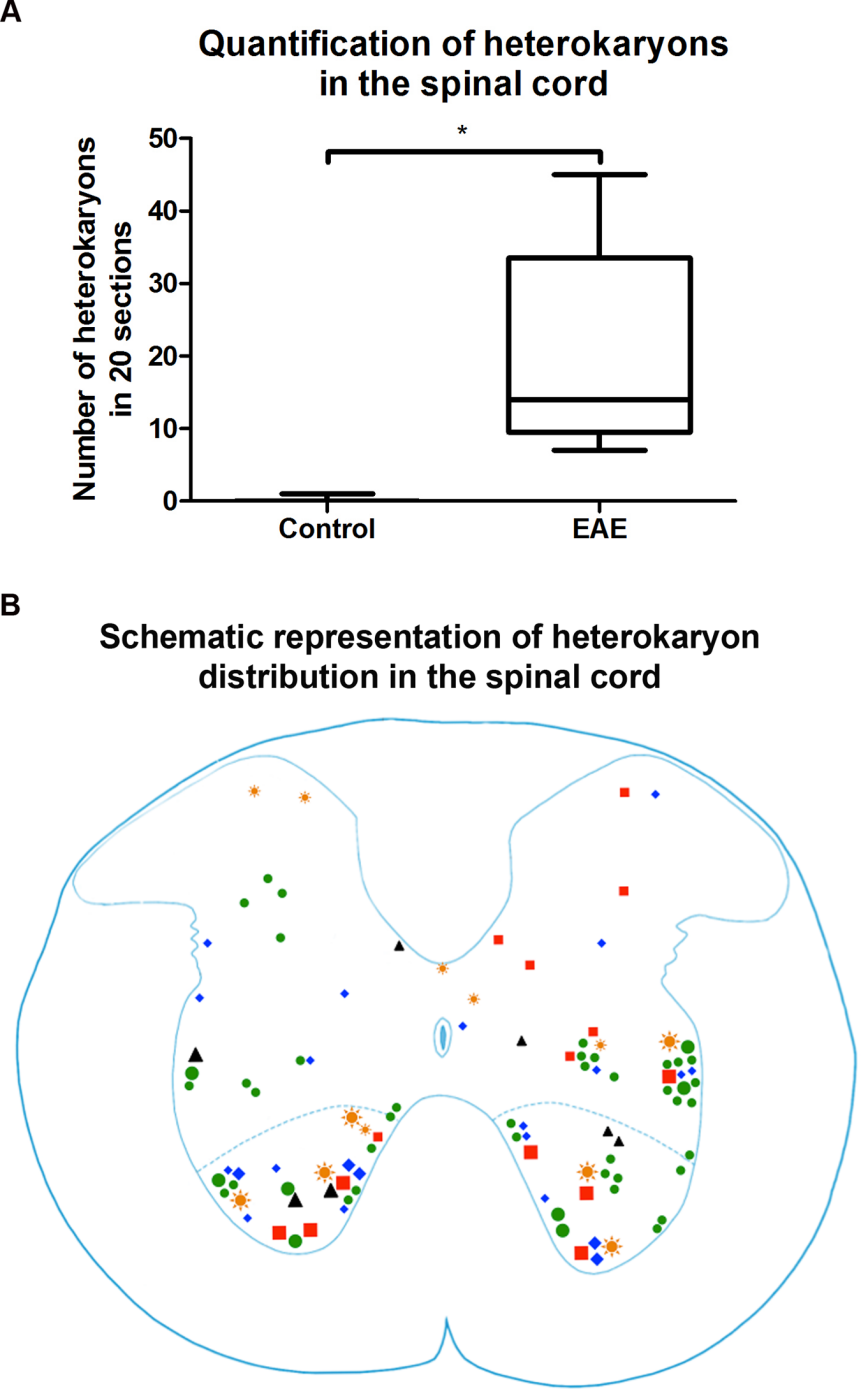
Quantification and distribution of heterokaryons in EAE affected and Control spinal cord. (A) While there was a wide spread in the number of heterokaryons between individual EAE affected mice (n = 5; 20.0 ± 6.7), depending on the severity of inflammation, there were significantly more heterokaryons (p = 0.0358, Mann-Whitney-Wilcoxon test) in EAE affected mice than Control (n = 3; 0.3 ± 0.3) mice. (B) Schematic representation of the distribution of heterokaryons in 20 sections of EAE spinal cord. Each symbol represents one experimental animal, and the symbol size represents heterokaryon size (small symbol: <20 μm, large: >20 μm).

**Fig 6:**
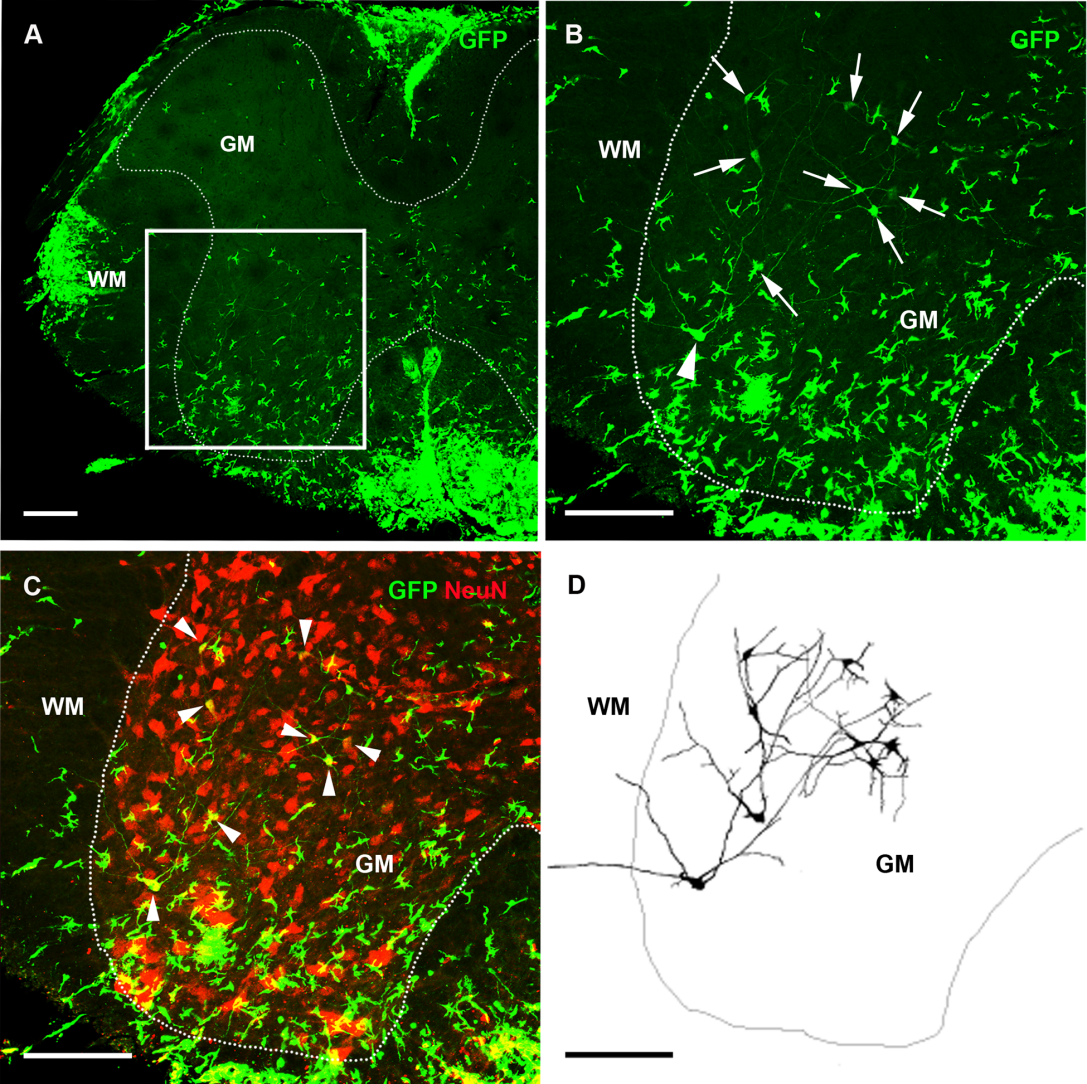
GFP-labeled motor neurons and interneurons in the spinal cord. (A) In a coronal section of the spinal cord with prominent infiltration of GFP-labeled bone marrow-derived cells (green), a number of GFP-labeled interneurons were detected. (B) Magnification of the area indicated with a box in (A). Eight GFP-labeled interneurons (green, indicated by arrows) located in the grey matter (GM), and one motor neuron (arrowhead) extending an axon/dendrites across the white matter (WM). (C) Most of these cells co-label with NeuN (indicated by arrowheads). (D) Schematic illustration of GFP-labeled interneuron and their dendrites. Scale bar (A-D) 150 μm.

The number of heterokaryons in EAE affected mice (n = 5; 20.0 ± 6.7) was higher than in non-immunized control animals (n = 3; 0.3 ± 0.3), (p = 0.0358, Mann-Whitney-Wilcoxon test, Fig 5A). We observed both large GFP-labeled motor neurons and small GFP-labeled interneurons scattered around the spinal cord (Fig 5B). Some of these GFP-labeled interneurons also expressed, NeuN (Fig 6A–6C, S8A–S8C, S9A and S9C and S10A–S10C Figs). The GFP-labeled motor neurons as well as the GFP-labeled interneurons contributed to the local neuronal circuitry (Fig 6D).

## Discussion

We have demonstrated that BMDCs can fuse with a broader repertoire of neurons in the CNS than previously demonstrated. We speculate that a yet unknown mechanism that requires the presence of inflammatory agents, and/or compromised neurons triggers cell fusion between BMDCs and neurons. This phenomenon is well described in large neurons such as Purkinje cells and in this study, could surprisingly also be reported for large spinal cord motor neurons. In contrasting to our previous hypothesis that cell fusion might be limited to large neurons, we now speculate that the same phenomenon also occurs in smaller interneurons in the spinal cord. Furthermore, we could not detect any heterokaryons in other areas of the CNS, including the retina, olfactory bulb, cortex, lateral ventricles or hippocampus. In our model, these heterokaryon-free areas were not affected by EAE and massive immune cell infiltration, supporting our and others’ hypothesis that cell fusion may be a rescue mechanism for compromised neurons [29].

The formation of heterokaryons in the CNS (cell fusion between transplanted GFP-labeled BMDCs and neurons) has been challenged by others due to the difficulties to demonstrate that these GFP-labeled heterokaryons are binucleate [17]. In this study, using a well described experimental set-up; we were able to show that some GFP-labeled motor neurons (Fig 3 and S4 Fig) and GFP-labeled interneurons were binucleate (S11 Fig). The few detected GFP-labeled cells that were binucleate may be due to the absence of the complete cell body in the sections or that alternative mechanisms could explain the presence of GFP in motor neurons and interneurons. We favor cell fusion based on our and other published results [5,15,16,18] but we cannot rule out other mechanisms such as (i) differentiation/transdifferentiation of BMDCs into neurons or (ii) transfer of foreign gene products (mRNA) through exosomes from BMDC into motor neurons. (i) We argue that it is improbable that BMDCs differentiate into motor neurons at the correct anatomical location, build a neuronal network as well as extending an axon into the sciatic nerve, (S7 Fig). (ii) Recently it was demonstrated that exosomes transferred Cre mRNA from BMDCs and induced recombination in Purkinje cells [30]. The recombined Purkinje cells expressed then GFP. In our model GFP is under the control of a ubiquitously expressing promoter (*chicken beta-actin*). If an exosome transferred GFP mRNA from a BMDC to a motor neuron, it could be argued that the GFP mRNA will be transcribed and the motor neuron would express GFP. However, it remains to be shown that mRNA or gDNA can be transferred by exosomes between two different cells and expressed/integrated over extended periods of time, this is an unlikely accord. To current evidence, given mRNA stability and GFP half-life versus our experimental design (transplanted ubiquitously expressing GFP BMDCs into wild-type recipients) cell fusion is much more probable. We further speculate that the cell soma of interneurons is so small that it is very difficult to discriminate between the two nuclei in GFP-labeled/NeuN-labeled interneurons. Furthermore, we cannot rule out nuclear fusion in these cells.

Recently, it was demonstrated that cell fusion also takes place between BMDCs and retinal neurons, but only when the retina was damaged. Fused hybrids were reprogrammed in a Wnt-dependent manner, and even contributed to functional restoration [29]. In our study, we did not detect any fusion in the retina. This is likely due to the fact that our model does not cause extensive damage and subsequent infiltration of immune cells into the retina. Yet, the study shows exciting evidence that under the right circumstances, cell fusion may contribute to regeneration in other areas in the CNS.

Cell fusion is commonly observed in a number of normal as well as pathological conditions. However, it is also detected in tumors, raising the concern that cell fusion between BMDCs and neurons may cause cancer. In our model, we never detected any abnormal heterokaryons yet alone-localized tumor formation; rather they resemble normal Purkinje cells or spinal cord neurons. Since neurons are post-mitotic, we argue that in the post-fusion state, the BMDC nucleus also deactivates mitotic programs. Sanges et al. recently reported that if BMDCs were not reprogrammed following fusion with retina neurons, they underwent apoptosis [29]. This indicates that cell fusion and formation of heterokaryons may constitute a safe therapy in the future, but we cannot rule out that it may cause cancer under specific conditions and this aspect needs further investigation.

Further studies are in progress to identify which subpopulation(s) within the bone marrow compartment fuses with neurons. Dissecting the mechanism underlying fusion between hematopoietic cells and neurons may open up a new avenue to induce cell fusion in a controlled manner. We propose that cell fusion and heterokaryon formation can be a promising novel therapy to restore compromised neurons in different neurodegenerative diseases including amyotrophic lateral sclerosis (ALS).

## Conclusions

The current findings suggest that heterotypic cell fusion with BMDCs is not restricted to large neurons or to the cerebellum, but can also occur with motor neurons and with small interneurons in the adult mouse spinal cord in response to stress to the immune system. This exciting observation opens up for further exploration of cell fusion in a regenerative context, and hopefully the findings can be extended to humans, where undoubtedly further studies are warranted.

## Supporting Information

S1 Fig Immune cell infiltration in the olfactory bulb. (A) GFP-labeled bone marrow-derived cells (green) infiltrate the olfactory bulb of Control and EAE affected animals alike. Scale bar, 100 μm. (B) Magnification of (A), the morphology of GFP-labeled bone marrow-derived cells infiltrating the olfactory bulb resembles that of monocytes or macrophages. Scale bar, 25 μm. (C) Rare GFP-labeled cells co-labeled with neuronal marker Map2 (red), a second nucleus could not be detected. Scale bar 25 μm.
(TIF)

S2 Fig Bone marrow-derived cells fuse with spinal cord motor neurons. (A) A putative spinal cord motor neuron that is present in the ventral horn of the spinal cord expresses GFP (green) as a result of fusion between a GFP expressing bone marrow-derived cell and a motor neuron. The arrowheads show a single green axon extending from the grey matter to white matter of the ventral horn of the spinal cord. (B) Higher magnification of a putative spinal cord heterokaryon shown in (A). Scale bars (A) 300 μm, (B) 25 μm.
(TIF)

S3 Fig Bone marrow-derived cell forming a heterokaryon with a spinal cord motor neuron. (A) Massive infiltration of GFP-labeled immune cells (green), in the spinal cord. A GFP-labeled motor neuron is present (arrow) in the right ventral part. (B-D) The same motor neuron as in A is shown, (B) co-expressing GFP (green) and (C) NeuN (red). (D) Co-localization of GFP and NeuN. (E) NeuN staining in the same section as (A). (F-H) Higher magnification of the section in (B-D) showing a motor neuron co-expressing GFP (green) and NeuN (red), which is surrounded by infiltrating inflammatory cells. Scale bar (A-E) 300 μm, (F-H) 150 μm.
(TIF)

S4 Fig Binucleated GFP-labeled motor neuron in the spinal cord. (A) Arrow indicates the position of a GFP-labeled motor neuron in the spinal cord. (B) Higher magnification of the neuron from (A). Z-stack image showing two nuclei (Hoechst, red) within the same cell, marked with arrows. (C-H) Selected individual serial images of the z-stack in (B). The two nuclei are indicated by arrowheads in (D-H), the second nucleus appears in (G) and (H). Scale bar (A) 150 μm, (C-H) 25 μm.
(TIF)

S5 Fig Co-localization of NeuN and GFP in a spinal cord motor neuron. (A) GFP-labeled motor neuron (arrow), located in the ventral horn. (B) Z-stack image of the GFP-labeled neuron in (A), showing co-localization of GFP (green) and NeuN (red). (C) Higher magnification of the motor neuron in (A) shows GFP staining (green), indicating fusion with a GFP-labeled bone marrow-derived cell. (D) NeuN staining (red) in the same neuron shown in (C) and (E) shows merge images. (F-H) showing higher magnification of (C-E). Scale bar (A) 300 μm, (C-E) 75 μm, (B) and (F-H) 25 μm.
(TIF)

S6 Fig A GFP-labeled motor neuron in the ventral horn of the spinal cord. (A) The GFP-labeled motor neuron is located in the ventral horn (arrow) of the spinal cord. (B-D) Higher magnification of the motor neuron in (A) reveals that the GFP-labeled motor neuron (arrow) present in the motor neuron pool, co-localize the neuronal marker, NeuN (red). Scale bar 150 μm.
(TIF)

S7 Fig GFP expressing axon in the sciatic nerve. (A) A GFP-labeled axon (green) was detected in the sciatic nerve, indicating fusion between a spinal cord motor neuron and GFP-labeled bone marrow-derived cell. (B) Nuclear staining (Hoechst, blue) confirms the absence of infiltrating GFP-labeled immune cells in that area. Scale bars 100 μm.
(TIF)

S8 Fig Fusion between GFP-labeled bone marrow-derived cells and interneurons in the grey matter. (A-C) Arrowheads indicate co-localization of GFP (green) and NeuN (red) in interneurons, indicating fusion. Scale bar 150 μm.
(TIF)

S9 Fig Fusion of GFP-labeled bone marrow-derived cells with motor neurons and interneurons in the spinal cord. (A) A GFP-labeled motor neuron (arrow), with an extending axon or dendrite across the spinal cord. Inlay, spinal cord cross section, indicating the position of the shown GFP-labeled neuron (arrow), and a second large GFP-labeled neuron (arrowhead). (B) Schematic representation of the GFP-labeled neuron seen in (A). (C) A large GFP-labeled neuron (arrowhead), co-staining with NeuN (red, insert), surrounded by several smaller GFP-labeled interneurons (arrows). (D) Schematic representation of the neurons shown in (C) (GM—grey matter, WM—white matter, CC—central canal). Scale bar 150 μm.
(TIF)

S10 Fig GFP-labeled interneuron in the spinal cord. (A-C) A small neuron in the spinal cord co-expresses GFP (green) and NeuN (red), indicating fusion between a GFP-labeled bone marrow-derived cell and an interneuron. (D) Schematic representation of the indicated neuron showing an axon and dendrites extending from the cell body. Scale bars 150 μm.
(TIF)

S11 Fig GFP-labeled binucleate interneuron in the spinal cord. (A-P) Immunohistochemistry demonstrating different GFP-labeled interneurons at different locations in the spinal cord. (A-D) A GFP-labeled neuron co-expressing the neuron marker ChAT (red), (E-H) GFP-labeled neuron with two nuclei (Hoechst, red). (I-L) Same cell as in (E-H) but with a different optical layer from the Z-stack. (M-P) GFP-labeled interneuron co-expressing the neuronal marker, NeuN (red) stained with the nuclei dye, Hoechst (blue).
(TIF)
