# Supplementary figures and images for: Cell Fusion along the Anterior-Posterior Neuroaxis in Mice with Experimental Autoimmune Encephalomyelitis
Source: PLoS One. 2015 Jul 24;10(7):e0133903. doi: 10.1371/journal.pone.0133903 (PMC4514791; doi:10.1371/journal.pone.0133903)

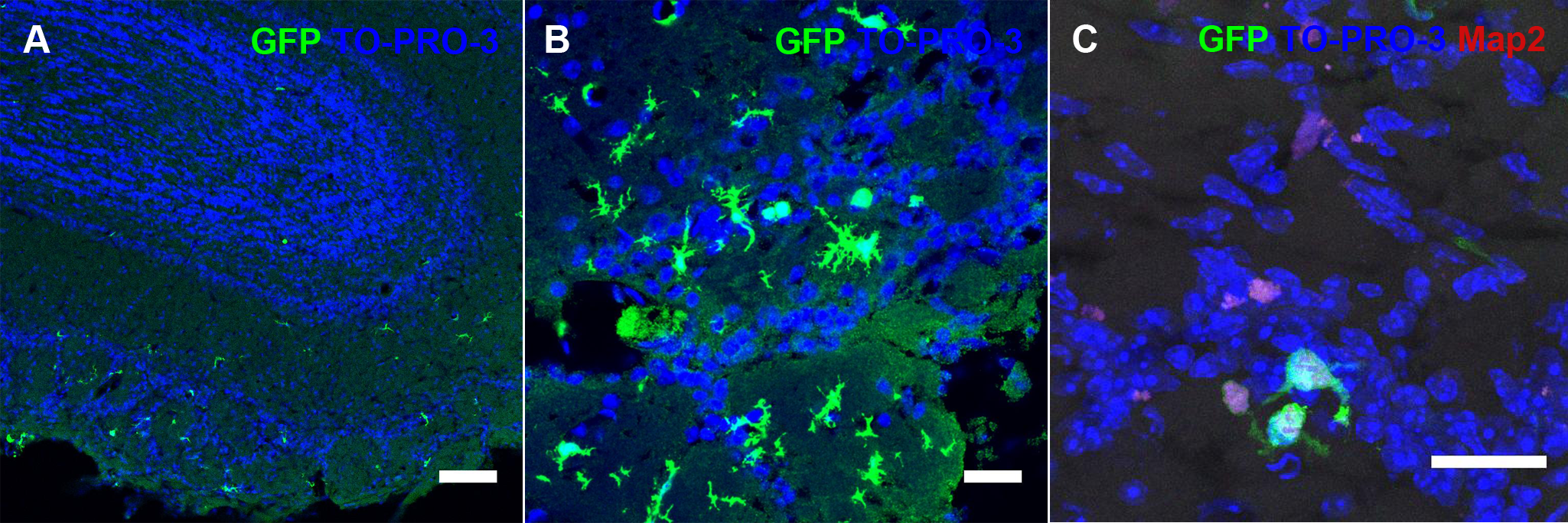

Supplement: S1 Fig — (A) GFP-labeled bone marrow-derived cells (green) infiltrate the olfactory bulb of Control and EAE affected animals alike. Scale bar, 100 μm. (B) Magnification of (A), the morphology of GFP-labeled bone marrow-derived cells infiltrating the olfactory bulb resembles that of monocytes or macrophages. Scale bar, 25 μm. (C) Rare GFP-labeled cells co-labeled with neuronal marker Map2 (red), a second nucleus could not be detected. Scale bar 25 μm. (TIF) [file pone.0133903.s001.tif]

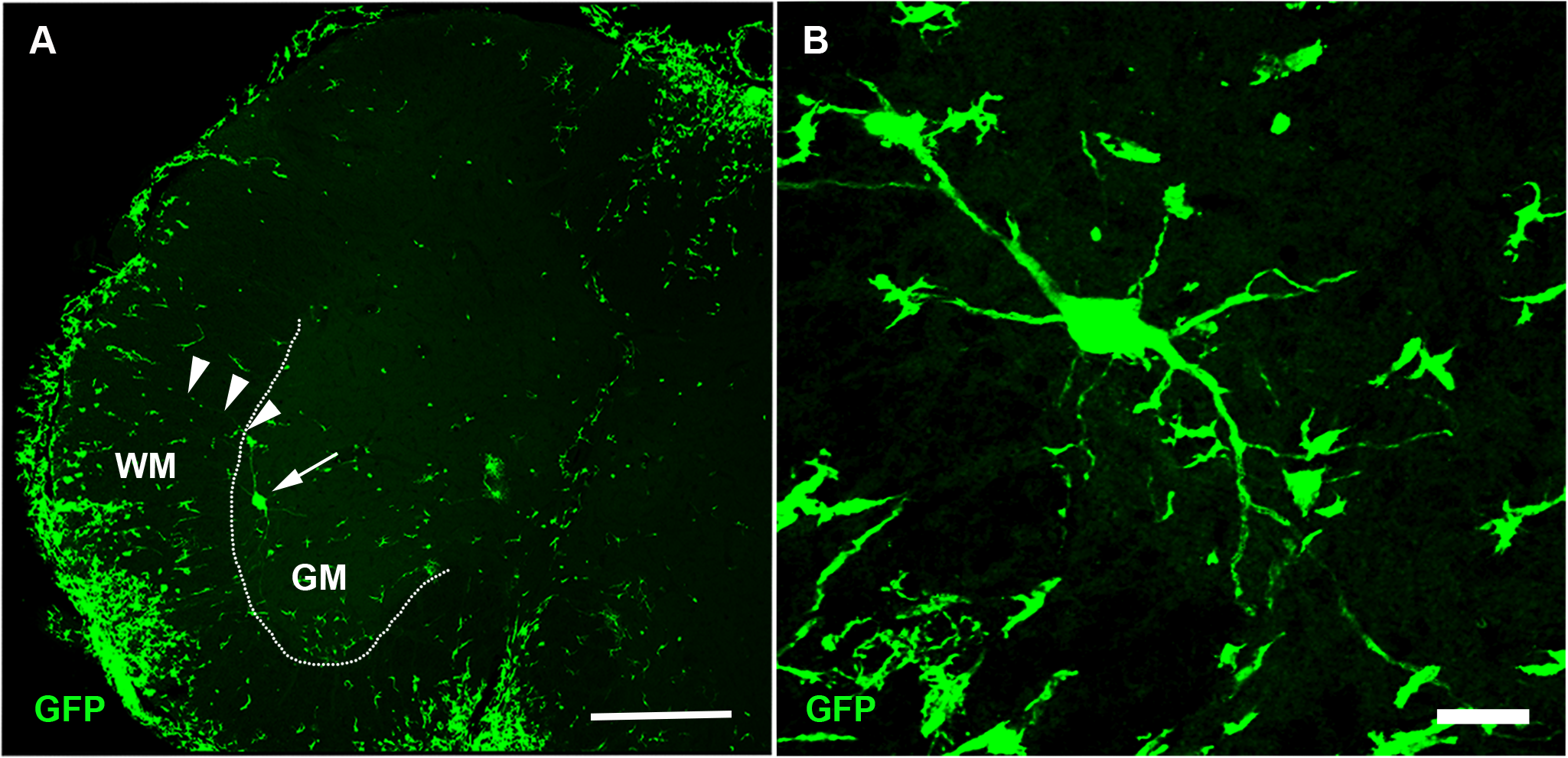

Supplement: S2 Fig — (A) A putative spinal cord motor neuron that is present in the ventral horn of the spinal cord expresses GFP (green) as a result of fusion between a GFP expressing bone marrow-derived cell and a motor neuron. The arrowheads show a single green axon extending from the grey matter to white matter of the ventral horn of the spinal cord. (B) Higher magnification of a putative spinal cord heterokaryon shown in (A). Scale bars (A) 300 μm, (B) 25 μm. (TIF) [file pone.0133903.s002.tif]

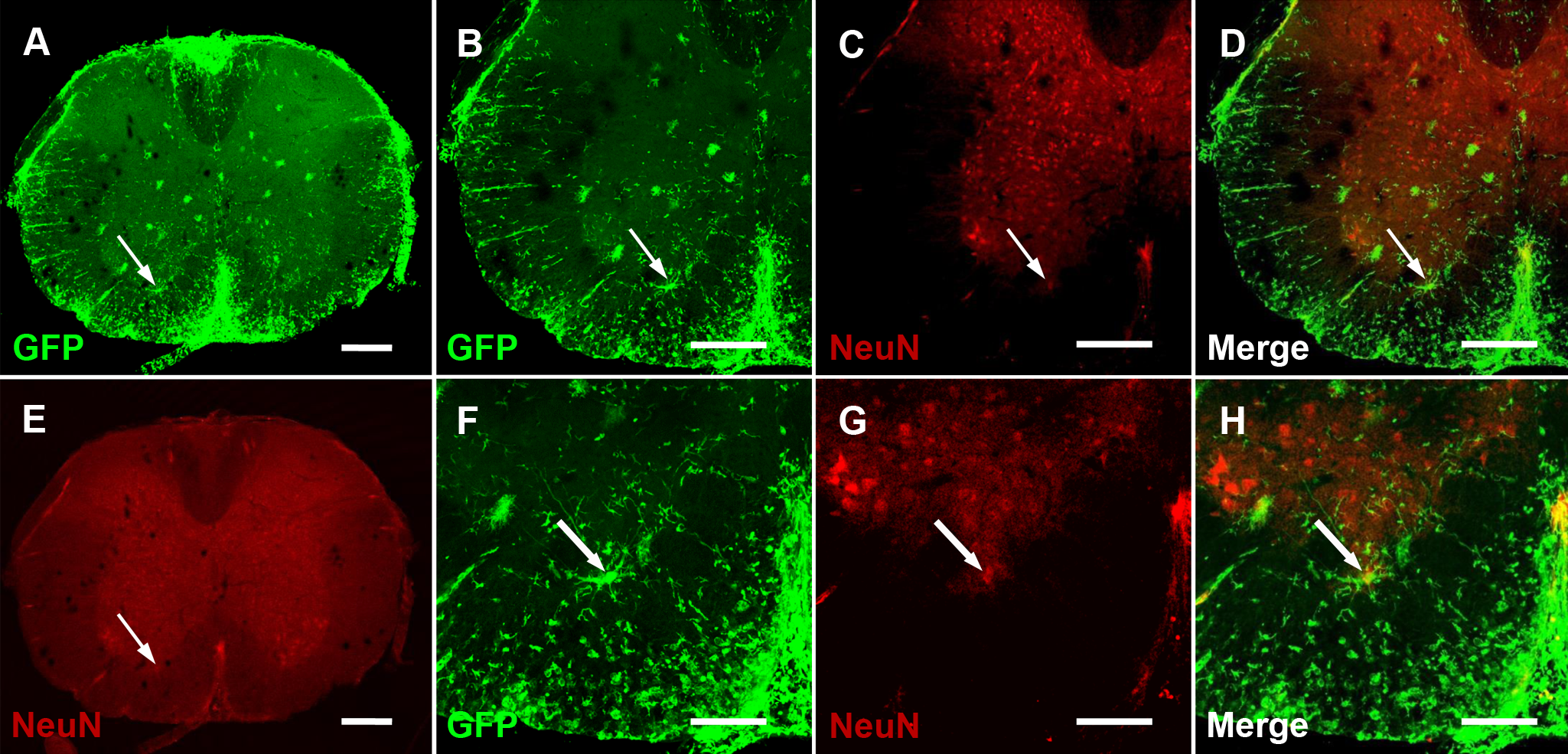

Supplement: S3 Fig — (A) Massive infiltration of GFP-labeled immune cells (green), in the spinal cord. A GFP-labeled motor neuron is present (arrow) in the right ventral part. (B-D) The same motor neuron as in A is shown, (B) co-expressing GFP (green) and (C) NeuN (red). (D) Co-localization of GFP and NeuN. (E) NeuN staining in the same section as (A). (F-H) Higher magnification of the section in (B-D) showing a motor neuron co-expressing GFP (green) and NeuN (red), which is surrounded by infiltrating inflammatory cells. Scale bar (A-E) 300 μm, (F-H) 150 μm. (TIF) [file pone.0133903.s003.tif]

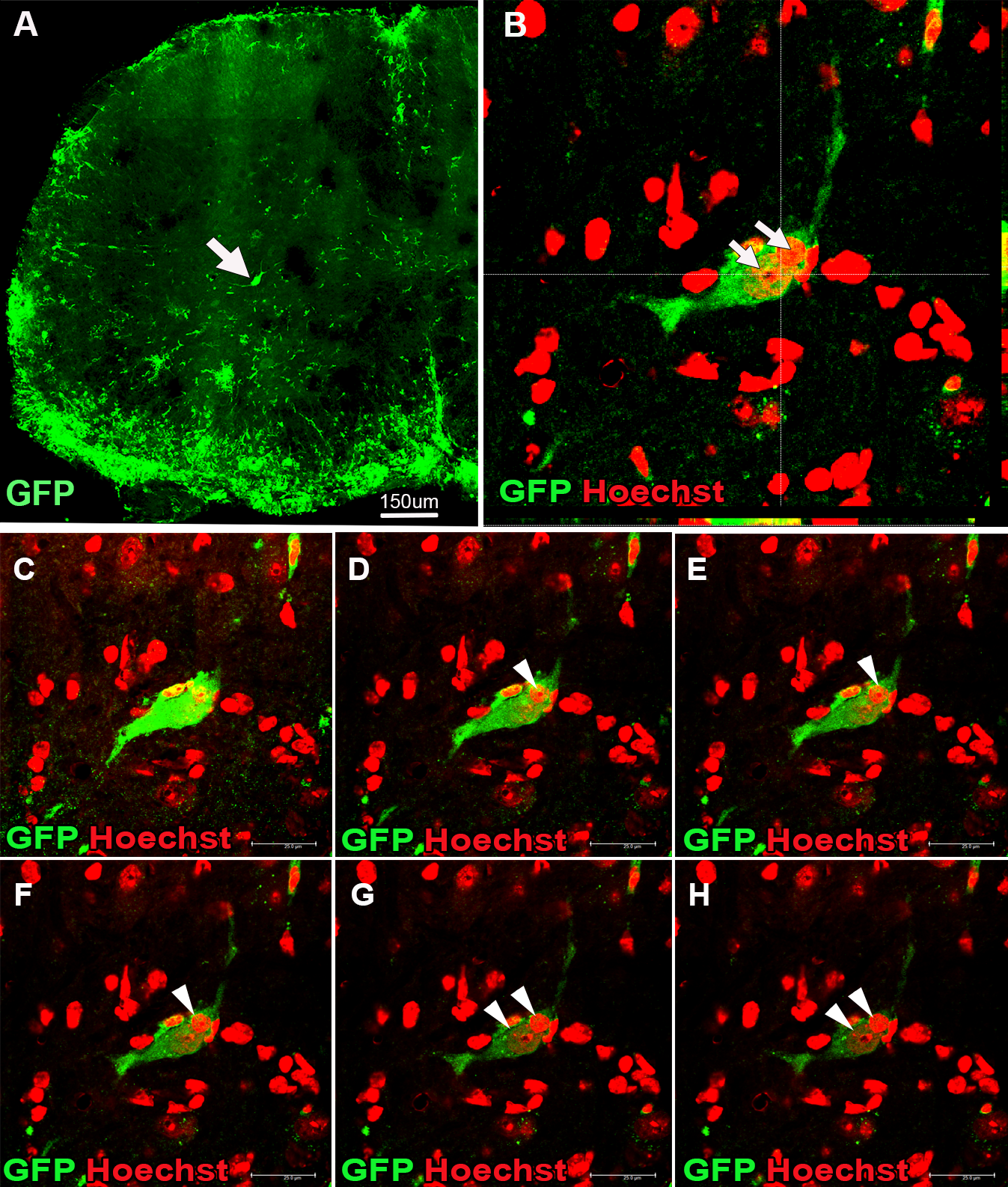

Supplement: S4 Fig — (A) Arrow indicates the position of a GFP-labeled motor neuron in the spinal cord. (B) Higher magnification of the neuron from (A). Z-stack image showing two nuclei (Hoechst, red) within the same cell, marked with arrows. (C-H) Selected individual serial images of the z-stack in (B). The two nuclei are indicated by arrowheads in (D-H), the second nucleus appears in (G) and (H). Scale bar (A) 150 μm, (C-H) 25 μm. (TIF) [file pone.0133903.s004.tif]

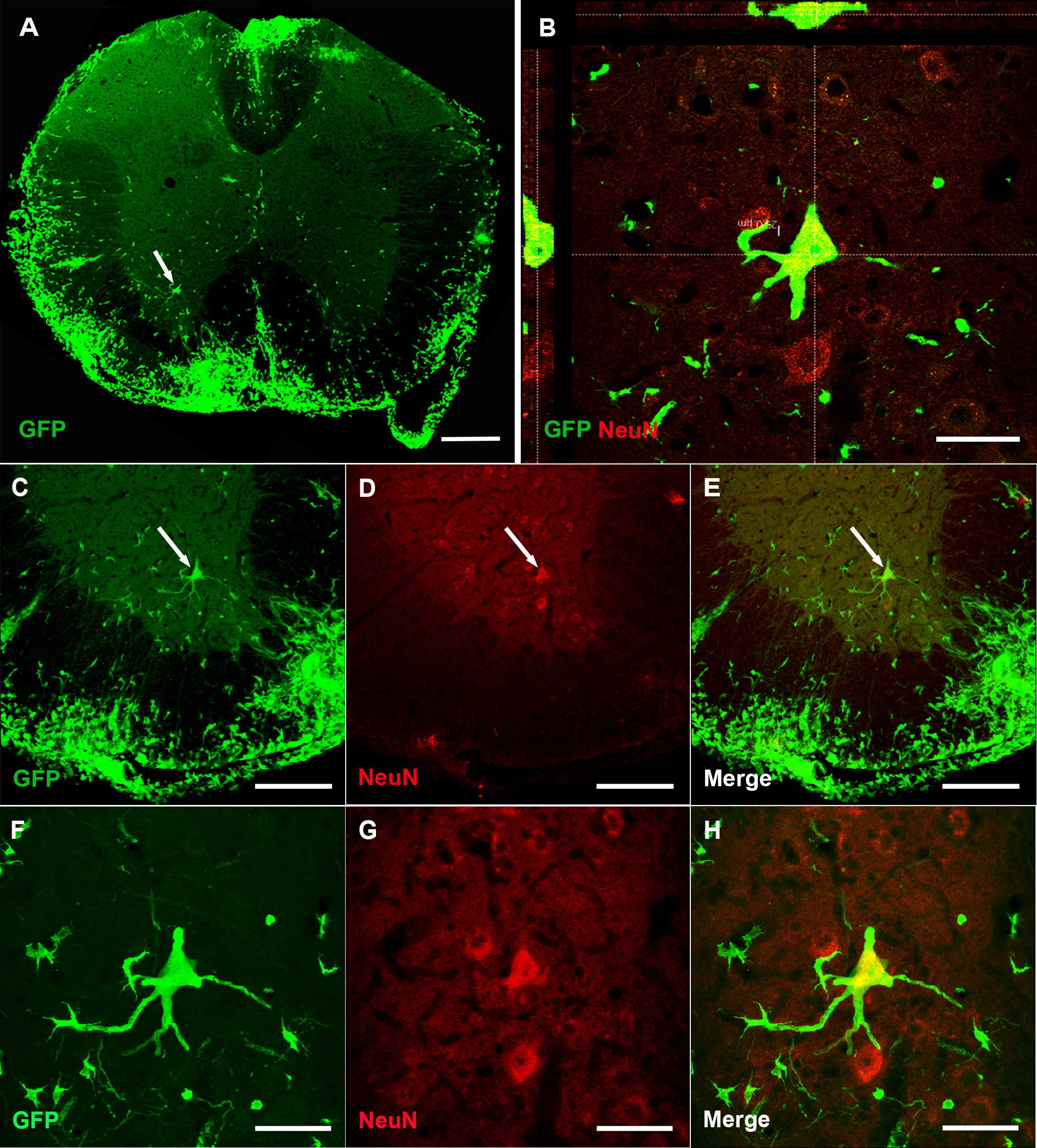

Supplement: S5 Fig — (A) GFP-labeled motor neuron (arrow), located in the ventral horn. (B) Z-stack image of the GFP-labeled neuron in (A), showing co-localization of GFP (green) and NeuN (red). (C) Higher magnification of the motor neuron in (A) shows GFP staining (green), indicating fusion with a GFP-labeled bone marrow-derived cell. (D) NeuN staining (red) in the same neuron shown in (C) and (E) shows merge images. (F-H) showing higher magnification of (C-E). Scale bar (A) 300 μm, (C-E) 75 μm, (B) and (F-H) 25 μm. (TIF) [file pone.0133903.s005.tif]

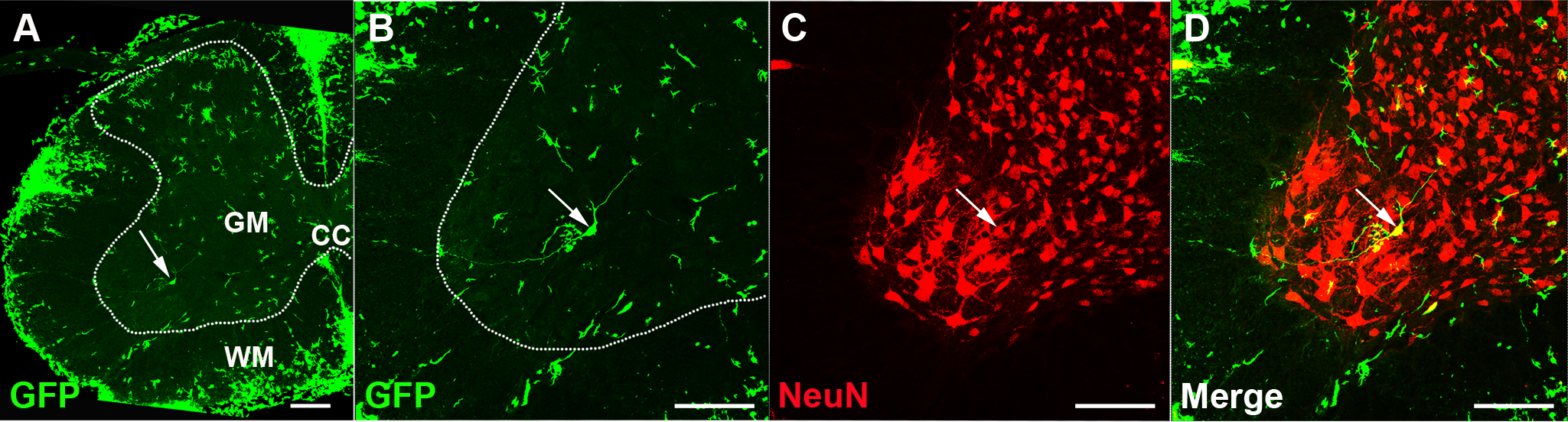

Supplement: S6 Fig — (A) The GFP-labeled motor neuron is located in the ventral horn (arrow) of the spinal cord. (B-D) Higher magnification of the motor neuron in (A) reveals that the GFP-labeled motor neuron (arrow) present in the motor neuron pool, co-localize the neuronal marker, NeuN (red). Scale bar 150 μm. (TIF) [file pone.0133903.s006.tif]

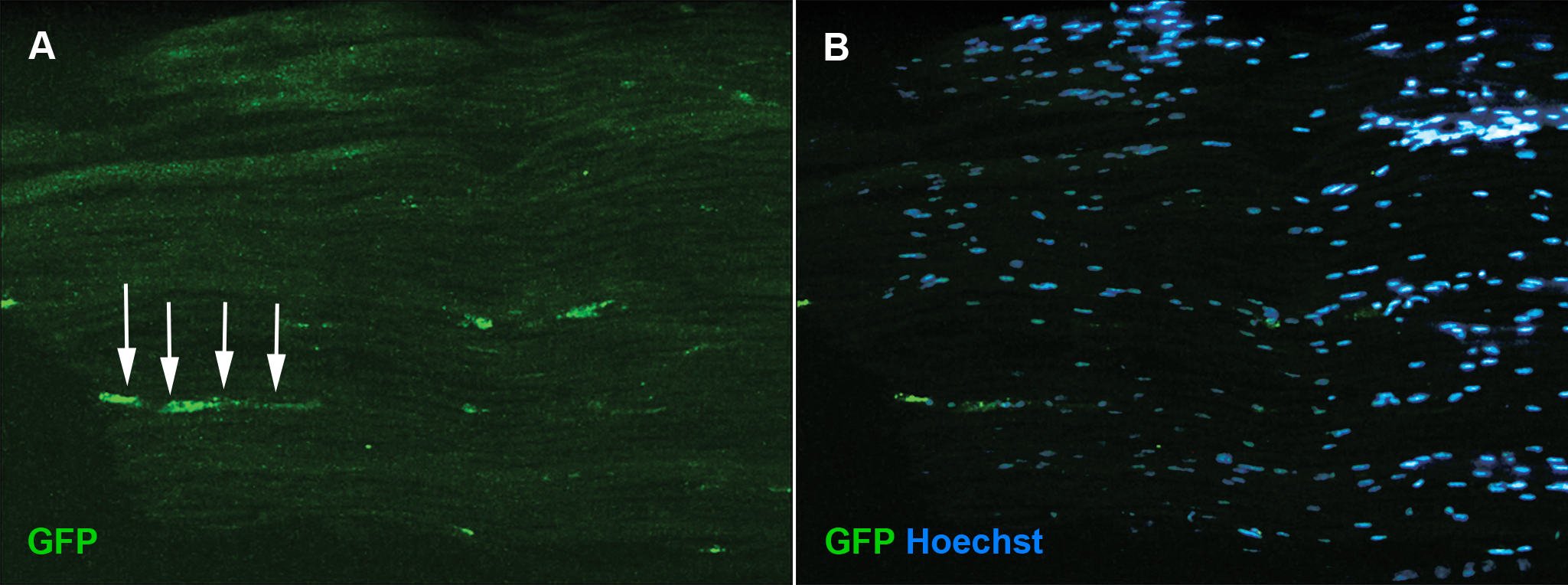

Supplement: S7 Fig — (A) A GFP-labeled axon (green) was detected in the sciatic nerve, indicating fusion between a spinal cord motor neuron and GFP-labeled bone marrow-derived cell. (B) Nuclear staining (Hoechst, blue) confirms the absence of infiltrating GFP-labeled immune cells in that area. Scale bars 100 μm. (TIF) [file pone.0133903.s007.tif]

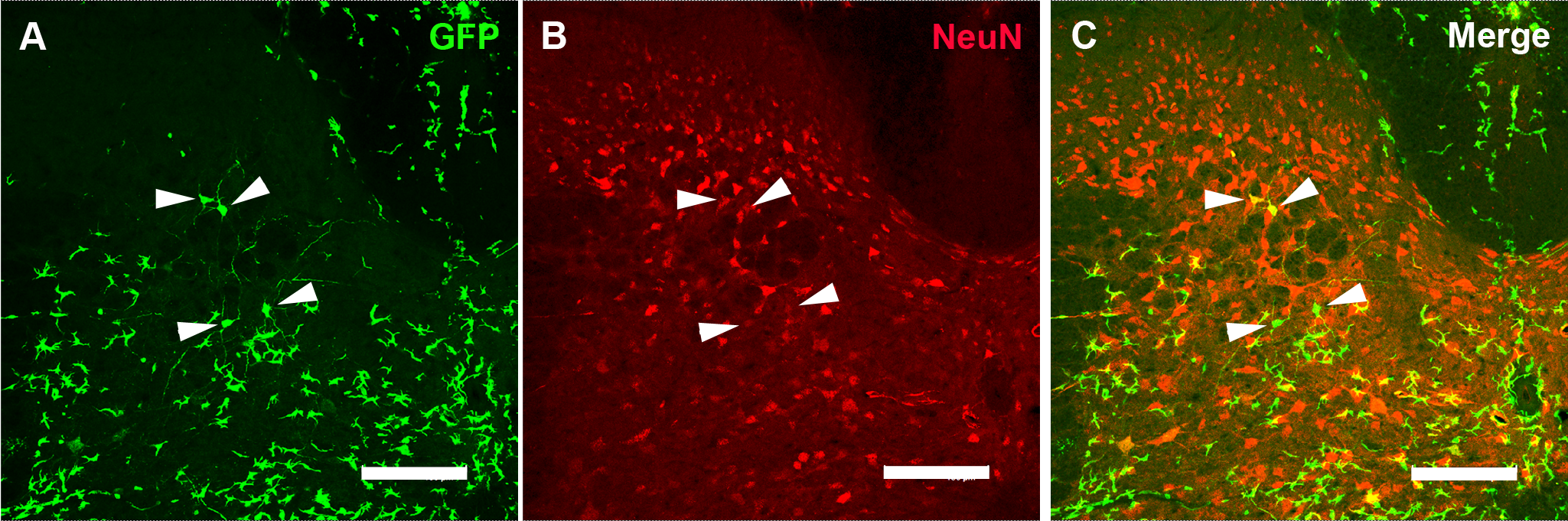

Supplement: S8 Fig — (A-C) Arrowheads indicate co-localization of GFP (green) and NeuN (red) in interneurons, indicating fusion. Scale bar 150 μm. (TIF) [file pone.0133903.s008.tif]

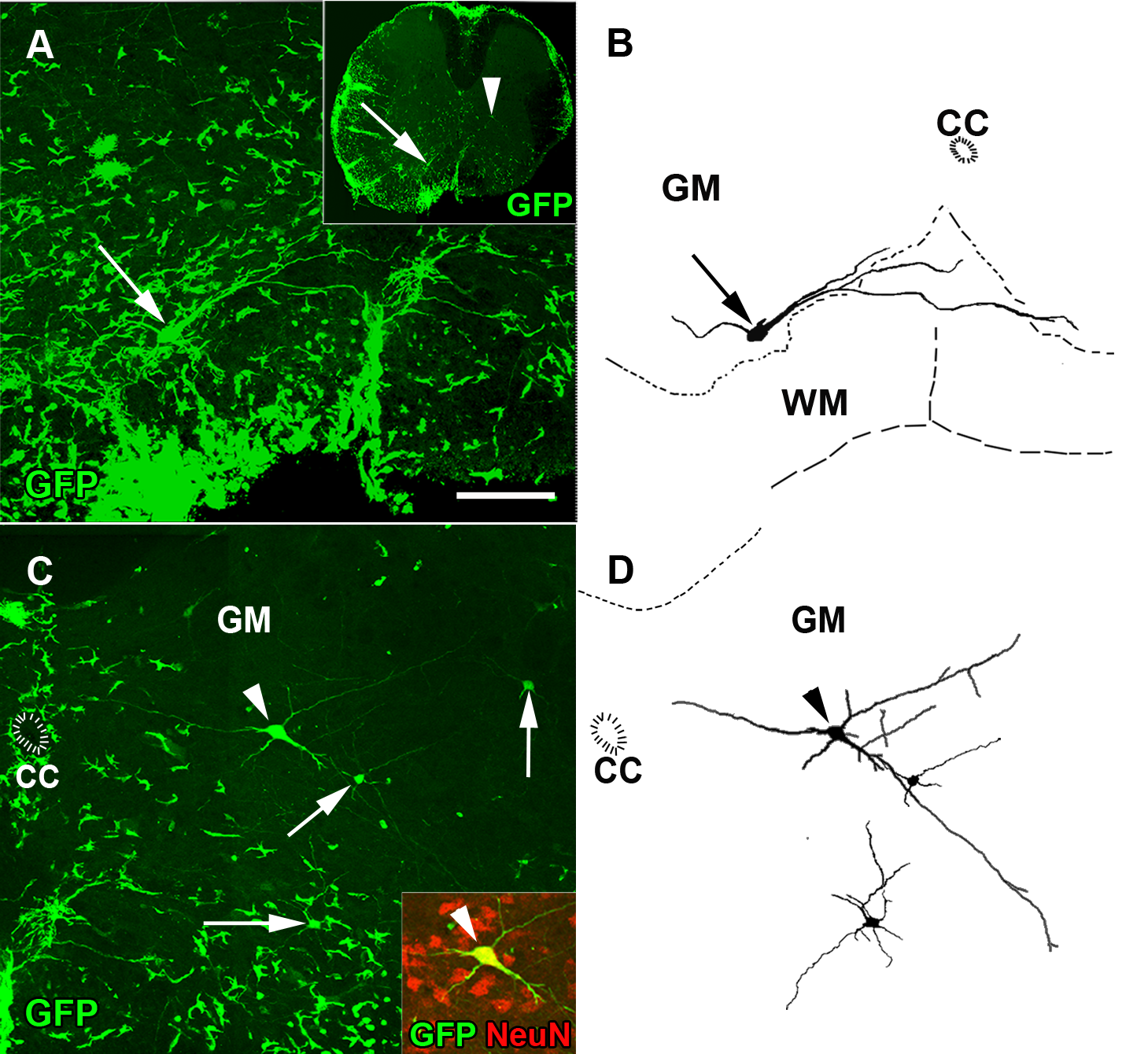

Supplement: S9 Fig — (A) A GFP-labeled motor neuron (arrow), with an extending axon or dendrite across the spinal cord. Inlay, spinal cord cross section, indicating the position of the shown GFP-labeled neuron (arrow), and a second large GFP-labeled neuron (arrowhead). (B) Schematic representation of the GFP-labeled neuron seen in (A). (C) A large GFP-labeled neuron (arrowhead), co-staining with NeuN (red, insert), surrounded by several smaller GFP-labeled interneurons (arrows). (D) Schematic representation of the neurons shown in (C) (GM—grey matter, WM—white matter, CC—central canal). Scale bar 150 μm. (TIF) [file pone.0133903.s009.tif]

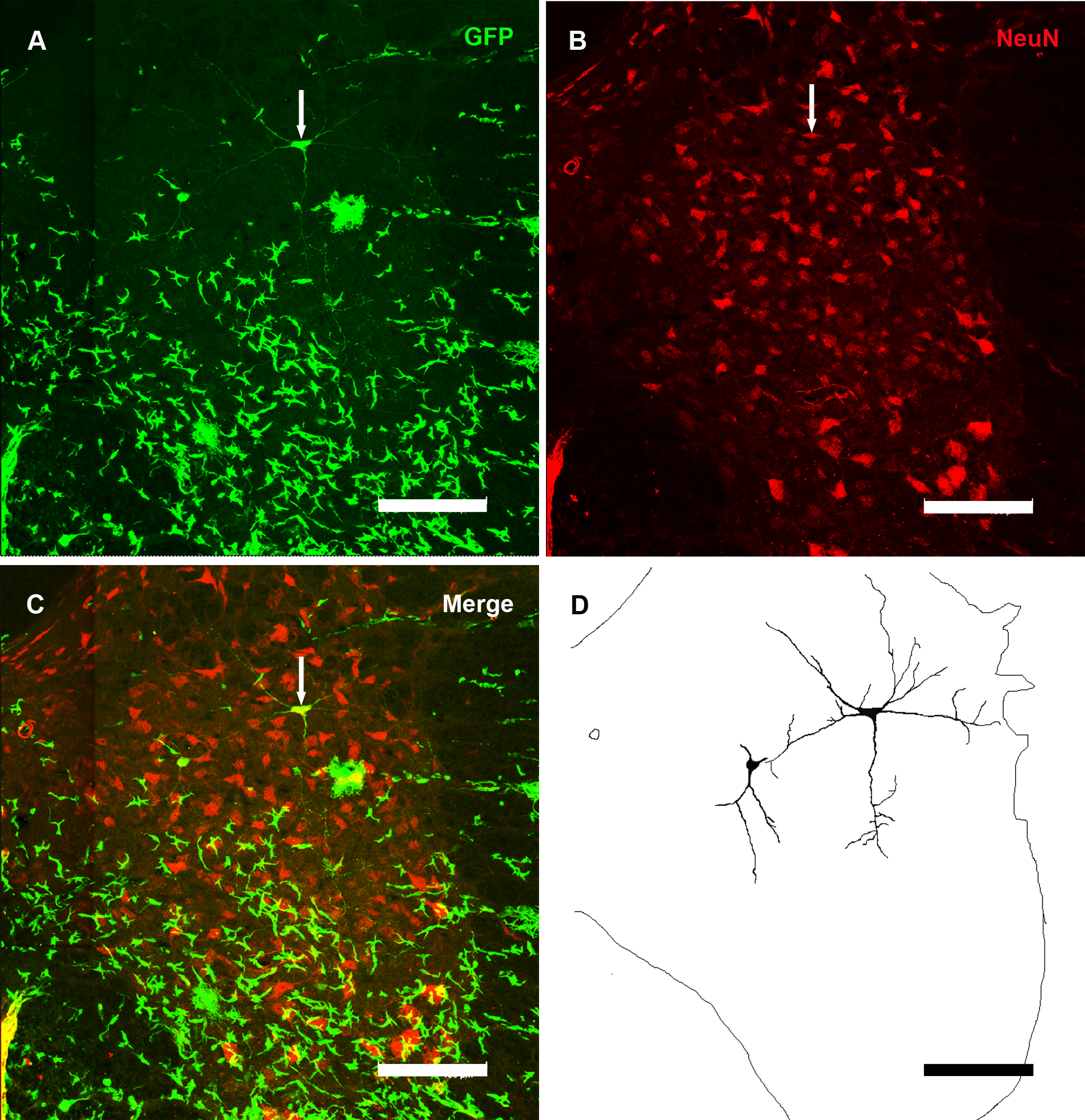

Supplement: S10 Fig — (A-C) A small neuron in the spinal cord co-expresses GFP (green) and NeuN (red), indicating fusion between a GFP-labeled bone marrow-derived cell and an interneuron. (D) Schematic representation of the indicated neuron showing an axon and dendrites extending from the cell body. Scale bars 150 μm. (TIF) [file pone.0133903.s010.tif]

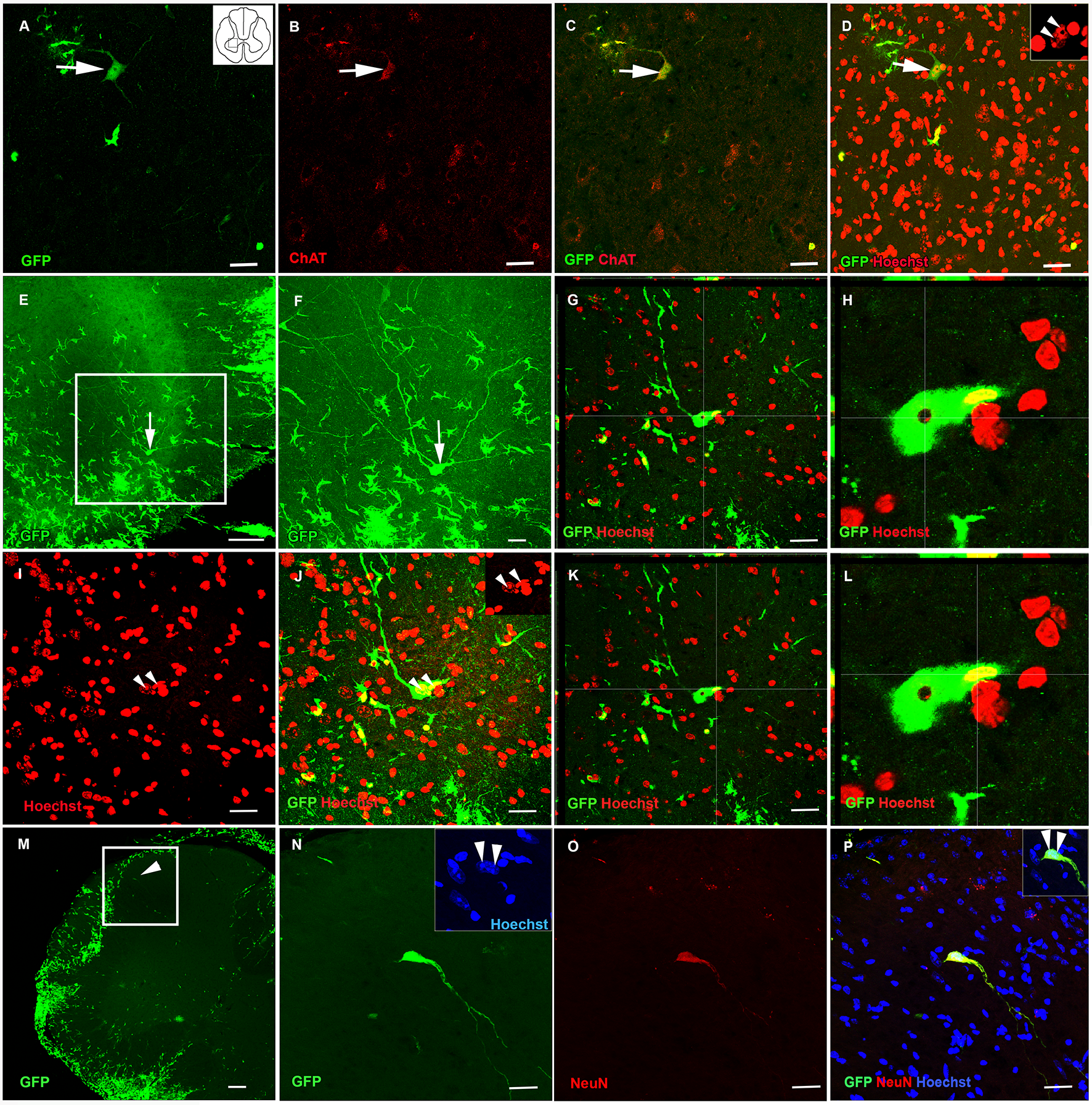

Supplement: S11 Fig — (A-P) Immunohistochemistry demonstrating different GFP-labeled interneurons at different locations in the spinal cord. (A-D) A GFP-labeled neuron co-expressing the neuron marker ChAT (red), (E-H) GFP-labeled neuron with two nuclei (Hoechst, red). (I-L) Same cell as in (E-H) but with a different optical layer from the Z-stack. (M-P) GFP-labeled interneuron co-expressing the neuronal marker, NeuN (red) stained with the nuclei dye, Hoechst (blue). (TIF) [file pone.0133903.s011.tif]
